# Implementation of Transabdominal Preperitoneal Repair in a Super-aged Region: A Propensity Score-Matched Analysis of Inguinal Hernia Repair in Patients Aged ≥75 Years

**DOI:** 10.7759/cureus.95700

**Published:** 2025-10-29

**Authors:** Koki Miya, Takeshi Kato, Fumitaka Yagi, Takashi Saitoh, Katsuhiko Suzuki

**Affiliations:** 1 Department of General Surgery, Honjo Daiichi Hospital, Yurihonjo, JPN

**Keywords:** inguinal hernia, open repair, propensity score matching, super-aged region, transabdominal preperitoneal repair

## Abstract

Purpose

This study aimed to compare the laparoscopic transabdominal preperitoneal repair (TAPP) and open repair in older patients with inguinal hernia and determine whether the TAPP approach offers superior safety and effectiveness.

Methods

We retrospectively reviewed the medical records of patients aged ≥75 years who underwent primary unilateral inguinal hernia repair at Honjo Daiichi Hospital, Akita Prefecture, Japan, between October 2016 and December 2024. Bilateral, recurrent, and emergency cases were excluded. Propensity score matching with age, sex, body mass index, American Society of Anesthesiologists physical status, and antithrombotic use yielded 27 patients each for the TAPP and open groups. Endpoints were operative time, blood loss, hospital stay, analgesic use, complications (Clavien-Dindo grade ≥II), and recurrence.

Results

The TAPP group had a significantly longer operative time (102.6 ± 28.6 vs. 86.3 ± 19.2 min, p = 0.018), but lower blood loss (0 vs. 2.5 g, p < 0.001) and a shorter hospital stay (4.37 ± 2.06 vs. 7.63 ± 2.72 days, p < 0.001) than the open group. Analgesic use (p = 0.297) and complication rates (p = 0.236) did not differ, and no recurrences were observed.

Conclusions

In our cohort of patients aged ≥75 years, the TAPP approach was associated with reduced blood loss and shorter hospital stays, with comparable safety to that of the open repair. In a super-aged region such as the Akita Prefecture, these findings support the TAPP approach as a safe and effective standard option for older adults.

## Introduction

Inguinal hernia is a common surgical condition caused by defects in the abdominal wall of the groin. Age-related changes in connective tissue are considered to increase the risk of occurrence, and aging is regarded as one of the most important contributing factors [1]. In older patients, perioperative risks are elevated owing to comorbidities and reduced physiological reserve; therefore, the choice of surgical approach must balance safety and efficacy.

The transabdominal preperitoneal repair (TAPP) approach has become widely adopted as a minimally invasive technique for inguinal hernia repair. Compared with open repair, its reported advantages include reduced chronic pain, as well as the ability to repair bilateral or occult hernias simultaneously [2]. However, concerns remain regarding longer operative times, the requirement for general anesthesia, and potential postoperative complications, particularly in the older adult population [3].

Globally, the aging population is increasing. Japan is among the countries experiencing the most rapid demographic shift, with the Akita Prefecture having the highest aging rate in the nation [4, 5] and being one of the most super-aged regions worldwide. Therefore, Akita represents a uniquely ‘super-aged region,’ and the evaluation of surgical treatments in such a setting may provide important insights for optimizing surgical strategies in an aging society.

At our institution, the TAPP approach was introduced in 2016. Its use gradually expanded as surgeons became more experienced and confident with the technique, and older patients were increasingly included in the indication for TAPP. This clinical evolution prompted us to retrospectively evaluate the safety and feasibility of TAPP in the older adult population.

In this study, we aimed to compare the TAPP approach and open repair in patients with inguinal hernia aged ≥75 years at a regional core hospital in Akita Prefecture. Our primary goal was to evaluate the feasibility and safety of the TAPP approach in the older adult patients, rather than to establish it as a universal standard treatment for this population. Our findings may help clarify the clinical validity of the TAPP approach and inform optimal surgical decision-making for the older adult population.

## Materials and methods

Study design and patients

This retrospective observational study included older patients (aged ≥75 years) who underwent inguinal hernia repair at Honjo Daiichi Hospital, Akita Prefecture, Japan, between October 2016 and December 2024. The TAPP approach was introduced at our institution in 2016. Patients were retrospectively identified from the electronic medical records of the hospital. Eligible cases were limited to primary unilateral inguinal hernias. Exclusion criteria comprised the following cases: bilateral hernias, because operative time and postoperative outcomes are substantially affected, making direct comparison with unilateral cases challenging; recurrent hernias, because surgical outcomes are influenced by the initial procedure and scar tissue, leading to heterogeneity; and emergency cases, because bowel resection or non-use of mesh is sometimes required, which can prolong operative time and compromise comparability with elective cases. These exclusions may limit generalizability to the entire older population; however, they were considered appropriate to ensure a valid assessment of the safety and efficacy of elective inguinal hernia repair. The study was conducted in accordance with the Declaration of Helsinki and approved by the Ethics Committee of Honjo Daiichi Hospital (approval number: HH24-11-20). Informed consent was obtained through an opt-out process.

Surgical procedure

The TAPP procedure was performed either by surgeons experienced in laparoscopic surgery or under their supervision. The surgical approach was selected based on a comprehensive assessment of the patient’s general condition, surgical history, and activities of daily living. All TAPP procedures were performed using a three-port technique. The standardized steps included peritoneal incision, hernia sac management, dissection of the preperitoneal space, mesh placement for repair, and peritoneal closure. To ensure consistency among surgeons, the same instruments and a BARD 3D Max Light Mesh (C. R. Bard, Inc., Murray Hill, NJ, USA) were used in all cases.

Open repair was mainly performed using the mesh-plug method (Rutkow-Robbins technique). Although mesh-plug repair is not the standard open technique recommended by the European Hernia Society (EHS) guidelines [2], it has been historically used in Japan and remains practiced in some institutions. According to the Japanese Hernia Society (JHS) Guidelines 2024, there are no significant differences in outcomes between the mesh-plug and Lichtenstein techniques, and it is recommended that surgeons perform the procedure with which they are most experienced [6]. All procedures were carried out under general anesthesia. The standardized steps included inguinal incision, hernia sac management, and mesh-plug insertion and fixation. To minimize inter-surgeon variability, the operations were performed or supervised by experienced surgeons, with the same surgical technique and materials used throughout.

Data collection

Collected variables included age, sex, body mass index (BMI), American Society of Anesthesiologists physical status (ASA-PS) classification, and use of antithrombotic therapy as independent variables. Data concerning operative time, intraoperative blood loss, postoperative length of stay, frequency of analgesic use, postoperative complications, and recurrence were collected as dependent variables. Operative time was defined as the interval from skin incision to skin closure. Estimated intraoperative blood loss (recorded in grams) was obtained from the anesthetic records documented by the attending anesthesiologist. Although minor variations in estimation are possible, these records are routinely used for quantitative assessment in our institution and are considered reliable for comparative analysis. Postoperative length of stay was defined as the number of days from surgery to hospital discharge. Analgesic use was assessed as the total number of as-needed analgesic administrations during hospitalization. Postoperative complications were defined as events occurring within 30 days of surgery and classified as Clavien-Dindo grade II or higher. Recurrence was defined as the reappearance of an inguinal hernia confirmed by physical examination or imaging during follow-up.

Propensity Score Matching

Propensity scores were calculated using logistic regression with age, sex, BMI, ASA-PS classification, and antithrombotic therapy as covariates. One-to-one matching was performed using a caliper width of 0.25.

Endpoints

Endpoints included operative time, intraoperative blood loss, postoperative length of stay, frequency of postoperative analgesic use, postoperative complications of Clavien-Dindo grade ≥II within 30 days, and recurrence rate.

Statistical analysis

Normality of continuous variables was assessed using a Shapiro-Wilk test. A Student’s t-test was applied for normally distributed variables, and a Mann-Whitney U test was used for non-normally distributed variables. Categorical variables were analyzed using χ² or Fisher’s exact tests, as appropriate.

To provide a more comprehensive interpretation beyond p-values, effect sizes were reported as mean differences or odds ratios (ORs) with corresponding 95% confidence intervals (CIs). For binary outcomes with zero cell counts, a Haldane-Anscombe correction was applied to calculate ORs and 95% CI.

No missing data were present, and all eligible patients were included in the final analysis.

A priori power analysis was not conducted owing to the retrospective design of the study. Given the limited sample size (27 vs. 27 after matching), the statistical power to detect differences, particularly for rare outcomes such as complications and recurrence, was limited. Therefore, non-significant findings should be interpreted with caution, as clinically meaningful differences may still exist. Statistical significance was defined as a two-sided p-value <0.05. Statistical analyses were conducted using SPSS (IBM Corp., Armonk, NY, USA) and R-Studio software, with the assistance of Editage (www.editage.com).

## Results

**Figure 1:**
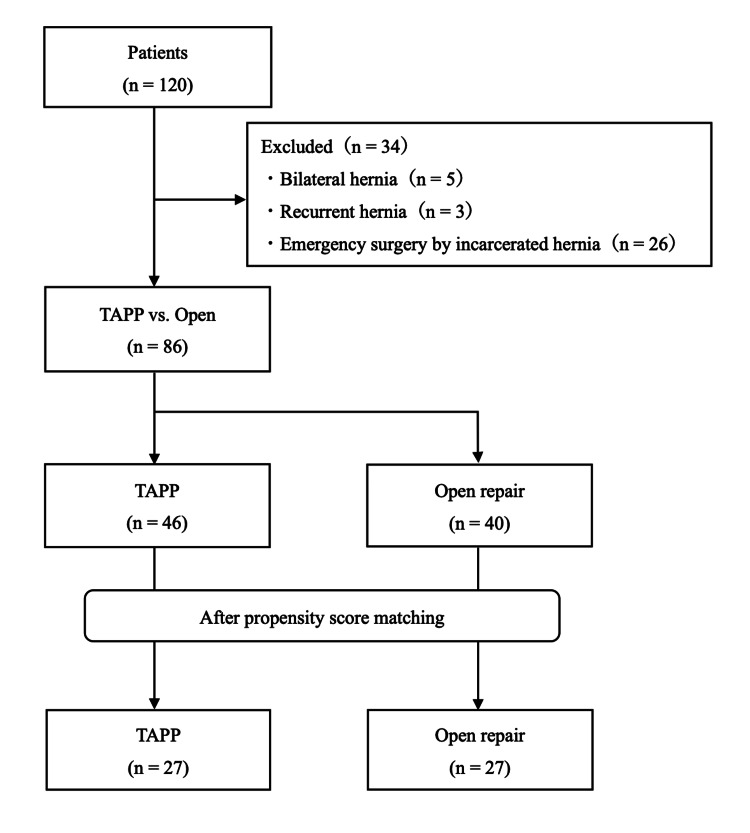
Flowchart of patient selection A total of 120 patients aged ≥75 years who underwent inguinal hernia repair were identified. Thirty-four patients were excluded; thus, 86 patients with primary unilateral inguinal hernia were eligible for analysis. After propensity score matching, 27 patients were assigned to each group. TAPP: transabdominal preperitoneal repair

Of the 120 cases identified, bilateral cases (n = 5), recurrent cases (n = 3), and cases of emergency surgeries for incarceration (n = 26) were excluded. Consequently, 86 patients (TAPP group, n = 46; open group, n = 40) were included in the analysis. After propensity score matching, 27 patients were assigned to the TAPP group and 27 to the open group (Figure 1).

Baseline characteristics

**Figure 2:**
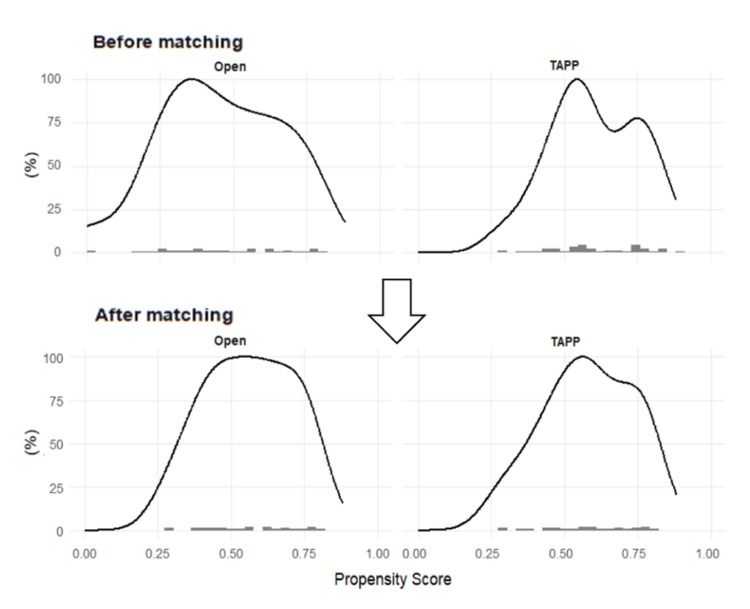
Propensity score distribution according to group: before and after matching The distributions of propensity scores in the TAPP and open repair groups are shown. Before matching, the distributions differed between the two groups. After propensity score matching, the distributions were well balanced, indicating improved comparability between the groups. TAPP: transabdominal preperitoneal repair

After matching, no significant differences were observed between the two groups in terms of age (81.5 ± 4.9 vs. 80.8 ± 5.4 years, p = 0.603), BMI (22.8 ± 2.8 vs. 22.9 ± 3.0, p = 0.933), ASA-PS classification (p = 0.889), sex (p = 0.311), or use of antithrombotic therapy (p = 0.750). Both groups were well balanced (Table 1, Figure 2).

**Table 1:** Comparison of clinical baseline data between TAPP and open repair Before matching, differences were observed in some baseline variables. After matching, no significant differences were found between the two groups in terms of age, BMI, sex, ASA-PS, or use of antithrombotic agents, indicating adequate balance between the groups. Data are presented as mean ± SD or n (%). Normality of continuous variables was assessed using a Shapiro–Wilk test. A student’s t-test was applied for normally distributed variables, and a Mann–Whitney U test was used for non-normally distributed variables. Categorical variables were analyzed using χ² or Fisher’s exact tests, as appropriate. ASA-PS: American Society of Anesthesiologists physical status; BMI: body mass index; TAPP: transabdominal preperitoneal repair

Variables		Before PSM			After PSM		
		TAPP (n = 46)	Open (n = 40)	p-value	TAPP (n = 27)	Open (n = 27)	p-value
Age (years)		80.5 ± 5.0	82.8 ± 5.5	0.024	80.8 ± 5.4	81.5 ± 4.9	0.603
BMI (kg/m²)		23 ± 2.8	21.5 ± 3.0	0.01	22.9 ± 3.0	22.8 ± 2.8	0.933
Sex	Male	37 (80.4%)	32 (80.0%)	0.96	20 (74.1%)	23 (85.2%)	0.311
	Female	9 (19.5%)	8 (20.0%)		7 (25.9%)	4 (14.8%)	
ASA-PS	Ⅰ	3 (6.5%)	3 (7.5%)	0.308	2 (7.4%)	3 (11.1%)	0.889
	Ⅱ	23 (50.0%)	23 (57.5%)		15 (55.6%)	14 (51.9%)	
	Ⅲ	20 (43.5%)	12 (30.0%)		10 (37.0%)	10 (37.0%)	
	Ⅳ	0	2 (5.0%)		0	0	
Antithrombotic agents		13 (28.3%)	9 (22.5%)	0.541	6 (22.2%)	7 (25.9%)	0.75

Endpoints

Table 2 lists the results obtained for all the endpoints. Compared with the Open group, the TAPP group showed a longer operative time, significantly lower blood loss, and significantly shorter postoperative hospital stay.

**Table 2:** Comparison of intraoperative and postoperative outcomes between TAPP and open repair The comparison of intraoperative and postoperative outcomes between TAPP and open repair groups after propensity score matching. Data are presented as mean ± SD or n (%). Normality of continuous variables was assessed using a Shapiro–Wilk test. A Student’s t-test was applied for normally distributed variables, and a Mann–Whitney U test was used for non-normally distributed variables. Categorical variables were analyzed using χ² or Fisher’s exact tests, as appropriate. These results suggest that, although the TAPP approach required a longer operative time, it was associated with reduced blood loss and a shorter hospital stay, while maintaining comparable safety. CI: confidence interval; TAPP: transabdominal preperitoneal repair

Variables		TAPP (n = 27)	Open (n = 27)	Effect size (95% CI)	p-value
operation time (min.)		102.56 ± 28.6	86.3 ± 19.2	+16.3 (95% CI: 2.95–29.6)	0.018
intraoperative blood loss (g)		0 [0–0]	2.5 [0–7.5]	–5.83 (95% CI: –9.27 to –2.40)	<0.001
postoperative length of stay (days)		4.37 ± 2.06	7.63 ± 2.72	–2.82 (95% CI: –4.71 to –0.92)	<0.001
analgesic use (times)		2.04 ± 1.91	2.67 ± 2.45	–0.63 (95% CI: –1.80 to 0.55)	0.297
Complications (Clavien-Dindo Classification)	Ⅱ	0	1 (3.7%)	OR 0.13 (95% CI: 0.006–2.59)	0.2358
	Ⅲa	0	2 (7.4%)		
recurrence		0	0	−	−

## Discussion

This retrospective study compared the TAPP approach with open repair in patients aged ≥75 years with inguinal hernia. While numerous studies have reported the efficacy and safety of laparoscopic inguinal hernia repair (TAPP repair), most of these investigations have included patients aged ≥60 or ≥65 years [3, 7- 10], and evidence focusing exclusively on patients aged ≥75 years remains limited [11, 12]. This study is therefore clinically and socially significant in demonstrating that the advantages of TAPP repair are preserved even in the older adult population.

Reduced blood loss and shorter postoperative hospital stay

In this study, the TAPP group showed significantly less intraoperative blood loss and a significantly shorter postoperative length of stay compared with the open group. The median blood loss was small in both groups (0 g in the TAPP group vs. 2.5 g in the open group) and did not represent clinically relevant hemorrhage; however, the statistically significant difference suggests that laparoscopic surgery is inherently less invasive. The magnified view afforded by laparoscopy allows for easier identification of vessels and more precise hemostasis. This minimally invasive nature, combined with earlier postoperative recovery and shorter hospitalization, supports the superiority of the TAPP approach. In a propensity score-matched analysis by Xu et al. [7], laparoscopic repair was independently associated with reduced blood loss and shorter length of stay, and their findings align with those in this study. Our study extends these findings by confirming that such advantages are also preserved in patients aged ≥75 years, a subgroup that has rarely been analyzed separately.

Although the postoperative hospital stay in this study was relatively longer than that typically reported in other studies, this difference likely reflects regional and healthcare system characteristics rather than surgical complications. Our hospital is located in a rural area of northern Japan, where outpatient access is limited compared with urban centers. Consequently, patients-particularly older adults-tend to remain hospitalized longer for wound observation and postoperative care to ensure safe discharge and continuity of recovery at home.

Prolonged operative time and its clinical implications

In this study, operative time was significantly longer in the TAPP group. Previous reports have shown no difference in operative time between the TAPP and open approaches [3, 7]; however, the TAPP approach involves multiple technical steps, including incision, dissection, and suturing, and is therefore influenced by surgeon proficiency and case complexity. Nevertheless, the TAPP approach offers the advantage of direct intraperitoneal visualization, which allows for accurate diagnosis. This is particularly beneficial in the detection and simultaneous repair of bilateral or occult hernias, where the TAPP approach may be more useful than open repair [13]. Such benefits are clinically meaningful in older adults, as they may reduce the risk of recurrence and the need for additional surgery. Moreover, in this study, despite the longer operative time, complication rates did not differ between groups, indicating that safety was not compromised. Thus, the prolonged operative time observed here can be considered clinically acceptable and may represent a transient phenomenon associated with the early phase of adopting the procedure.

Safety in older patients

In this study, a small number of Clavien-Dindo grade ≥II complications occurred in the open group, whereas none were observed in the TAPP group; however, the difference was not statistically significant. Previous studies have suggested that laparoscopic repair can be performed safely in older patients; nevertheless, most of these studies analyzed patients aged ≥60 or ≥65 years as a single group [3, 7- 10], and robust evidence specifically targeting those aged ≥75 years is extremely scarce [11, 12]. Among the limited reports available, Ciftci reported comparable complication rates and faster recovery in patients aged >75 years, while Zhu et al. [11-12] confirmed that laparoscopic repair can be safely performed in octogenarians without increasing morbidity or recurrence. However, these previous studies may have been affected by selection bias, as patients with higher surgical risk or poor general condition were less likely to undergo laparoscopic repair. Moreover, neither study adjusted for preoperative differences in physical status, such as the American Society of Anesthesiologists physical status (ASA-PS) classification, which can substantially influence perioperative outcomes. In contrast, the present study utilized propensity score matching to minimize these confounding effects, balancing important covariates, including ASA-PS and antithrombotic therapy, between groups. This methodological strength provides a more robust assessment of the safety and feasibility of TAPP in older adults. Therefore, the present study provides an important contribution by demonstrating that the TAPP approach can be safely performed even in the older population, reinforcing the applicability of minimally invasive surgery in this vulnerable population.

Aging in the Akita Prefecture and global significance

Japan is the most aged society in the world, with an aging rate exceeding 28%. The Akita Prefecture, in particular, has the highest proportion of older adults in Japan, with approximately 39% of the population aged ≥65 years, far surpassing the national average [4, 5]. Thus, Akita can be regarded as a region that represents a ‘foreshadowing of the world’s future.’ The findings of this study, derived from such a super-aged setting, provide insights that extend beyond local characteristics and may be universally applicable to countries that will face further aging in the coming decades.

Older patients frequently present with comorbidities and frailty, raising concerns about postoperative decline in activities of daily living and delayed return to society [3, 14]. The TAPP approach, which is minimally invasive and has the potential to facilitate early discharge, may serve as an effective strategy to address these challenges. Moreover, the ability to repair bilateral and occult hernias simultaneously reduces the likelihood of reoperation and contributes to the efficient use of limited healthcare resources.

In addition, the TAPP approach integrates multiple technical steps-incision, dissection, and suturing-and therefore has significant educational value in fostering fundamental laparoscopic skills. Brucchi et al. reported that the TAPP approach is useful for skill acquisition among young surgeons [15]. The introduction of the TAPP approach may improve clinical outcomes and contribute to securing the surgical workforce and establishing a sustainable surgical care system in the community through the training of junior surgeons.

Limitations

This study has some limitations. It was conducted at a single center with a retrospective design and a relatively small sample size, which limits the generalizability of the findings and statistical power to detect rare events. Consequently, rare complications, chronic pain, and long-term recurrence rates could not be fully assessed. Patient-reported outcomes and quality-of-life assessments were not included, which may be particularly relevant in older adults. The study was performed in a super-aged region in Japan, and generalizability to other healthcare systems may be limited. This study also includes cases from the early phase of our institutional experience with the TAPP approach, during which the learning curve may have influenced certain perioperative outcomes. Future multicenter prospective studies with larger cohorts and comprehensive evaluations, including health economic analyses, are warranted to validate and extend our findings.

## Conclusions

Compared with open repair, the TAPP approach for inguinal hernia repair in patients aged ≥75 years demonstrated advantages such as lower intraoperative blood loss and shorter postoperative hospitalization, while maintaining safety with respect to complications and recurrence. Consistent with previous reports, these favorable outcomes were preserved even in the older adult population. These findings were obtained in one of the most rapidly aging regions worldwide and suggest that the TAPP approach can serve as an effective and minimally invasive standard option regardless of patient age. Furthermore, from an educational perspective, the TAPP approach contributes to the development of laparoscopic skills among surgeons and may play a role in fostering surgical workforce capacity and establishing a sustainable healthcare system in regional settings.
